# Coronary Artery Bypass Graft in a Young Man

**DOI:** 10.7759/cureus.1317

**Published:** 2017-06-05

**Authors:** Stella Pak

**Affiliations:** 1 Internal Medicine, University of Toledo Medical Center

**Keywords:** coronary artery bypass graft (cabg), myocardial infarction (mi), young adults

## Abstract

Currently, there are no explicit guidelines on the management of acute myocardial infarction (MI) in young adults under the age of 30. The lack of published literature to guide clinicians in the care of young adults with MI has prompted us to report a case exemplary for the successful treatment with coronary artery bypass graft (CABG). A 25-year-old man developed acute MI and underwent on-pump five quintuple CABG using bilateral internal mammary arteries and left greater saphenous vein. This appropriate and timely intervention may have helped to prevent cardiac death and maintain the quality of life.

## Introduction

Framingham Heart Study reported the incidence of myocardial infarction (MI) to be 1.29% in men aged 30 to 34 years old and 0.52% in women aged 35 to 44 [1]. The incidence of MI in young adults is very rare but the ones necessitating coronary artery bypass graft (CABG) are even rarer [2]. Due to deficient research evidence specifically targeted towards younger population, there are no explicit guidelines on the management of acute MI in young adults. Acute MI can result in death, or significant physical, psychological, or social disabilities in young patients in the prime of their most productive years. Therefore, it is important to aggressively control risk factors for primary and secondary prevention, provide appropriate and timely management, and help those who suffered MI to return to pre-MI functionality. Herein, we report a case of MI in a young man successfully managed with an emergency CABG.

## Case presentation

A 25-year-old male presented to the Emergency Department with progressively worsening left-sided chest pain and dyspnea on exertion over the past four days. His medical history was notable for coronary artery disease and has had seven stents placed since the age of 18. He also had a history of systemic lupus erythematous (SLE), hypertension, hyperlipidemia, obesity, and end-stage renal disease (ESRD) on hemodialysis. Subsequent history revealed 11 pack years of smoking and noncompliance with medication regimens, hemodialysis schedule, and nutrition therapy. There was no family history of premature cardiovascular disease.

Cardiac enzyme values were as follows: troponin 0.05 ng/mL, creatine kinase 36 U/L, and CK-MB 2.2 ng/mL. Electrocardiogram (ECG) revealed ST depression in leads I, II, and V4-6. Cardiac enzyme values obtained about six hours later suggested the evolving nature of MI: 0.07 ng/mL, creatine kinase 33 U/L, and CK-MB 3.5 ng/mL. The last set of cardiac enzymes collected 18 hours from the initial presentation were troponin 1.12 ng/mL, creatine kinase 73 U/L, and CK-MB 15.9 ng/mL.

**Figure 1:**
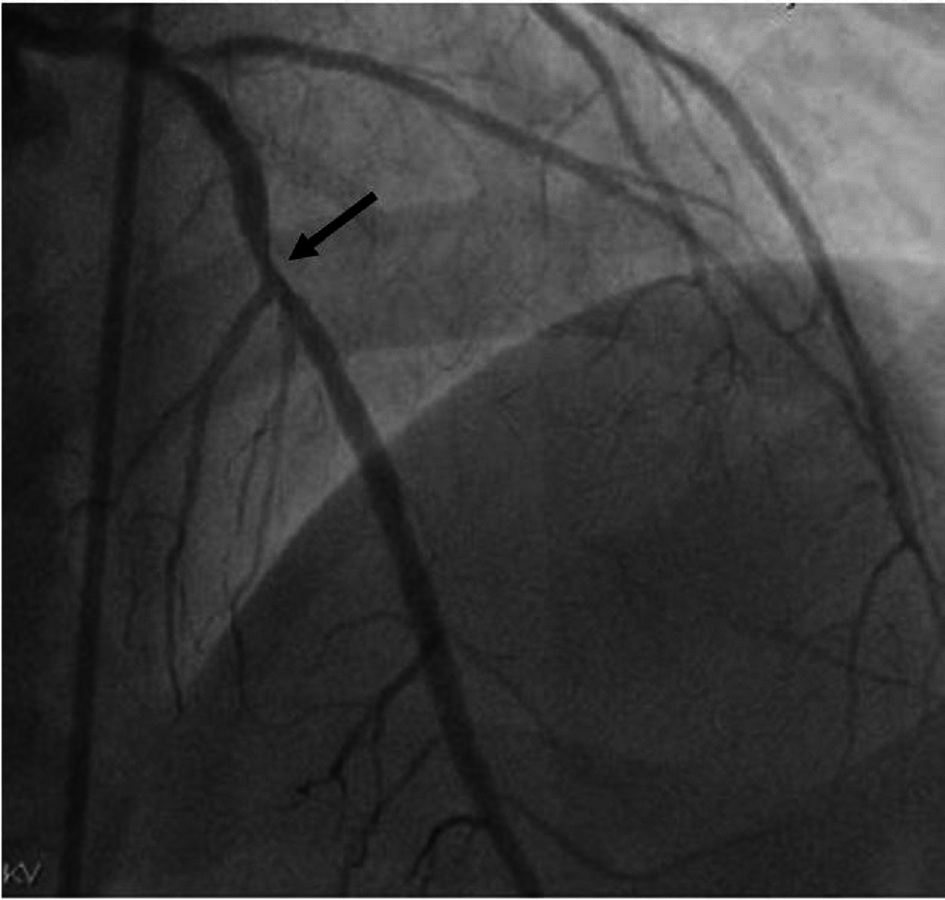
Coronary angiogram demonstrating 70 to 80% in-stent restenosis at the left anterior descending coronary artery.

Transthoracic echocardiogram (TTE) showed concentric left ventricular hypertrophy with left ventricular ejection fraction (LVEF) 55%. Coronary angiogram (Figure 1) revealed 70 to 80% in-stent restenosis at the left anterior descending coronary artery (LAD). Obtuse marginal branch of left circumflex coronary artery (LCX) also had a complex 90 to 99% stenosis with in-stent restenosis at the ostium. In the right coronary artery (RCA), 60 to 70% stenosis just distal to the posterior descending artery (PDA) was noted.

In view in-stent restenosis in multiple coronary vessels and evolving nature of MI, an urgent five-quintuple CABG was chosen over percutaneous intervention with stent placement. Bilateral internal mammary arteries (IMAs) and left greater saphenous vein (GSA) were harvested via median sternotomy and endoscopy, respectively. CABG consisted of left IMA to the main LAD, right IMA to the obtuse marginal branch of LCX and first diagonal branch of the LAD (with inflow into the RIMA graft from LIMA), and left GSA to the second diagonal branch of the LAD and the PDA of the RCA.

Following the operation, the patient was transferred to critical care unit in stable condition. The patient showed an uneventful postoperative course and was discharged on day 6 of hospital admission. At one-month follow-up, CT angiography confirmed patency of the grafts. The patient also reported complete resolution of angina and dyspnea.

## Discussion

The key culprits for acute MI in this patient are most likely to be non-modifiable risk factors, including SLE and ESRD. On the other hand, our patient had multiple modifiable risk factors for MI: hypertension, hyperlipidemia, obesity, smoking, noncompliance with medication regimens, hemodialysis schedule, and diet recommendation.

Vascular endothelial injury in SLE is mediated by immune complex deposition. This vascular insult sets the cascade of atherosclerosis in motion, which in turn, is accelerated by the circulating cytokines in systemic inflammation [3]. ESRD also contributes to the development of MI by promoting myocardial fibrosis, ventricular dilation, and hypertrophy [4]. These are non-modifiable, but controllable risk factors. Their effect could be reduced by consistent adherence with medication regimen, hemodialysis, and diet recommendation. However, poor therapeutic compliance unleashed rampant cardiac manifestation of the chronic diseases in our patient.

Of note, multiple modifiable factors were poorly managed in this high-risk profile patient. The modifiable factors include smoking, obesity, hypertension, and hyperlipidemia. This case highlights the need for aggressive management of risk factors in young adults. In young individuals, the medical, financial, and social burden from MI is amplified in terms of potentially productive years of life lost.

## Conclusions

In summary, we presented a case of evolving acute MI in a young man successfully treated with emergency CABG. We hope this exemplary case helps clinicians to properly care for multi-vessel MI in young patients.​
